# Applicability of Plant Extracts in Preclinical Studies of Melanoma: A Systematic Review

**DOI:** 10.1155/2018/6797924

**Published:** 2018-07-26

**Authors:** Kamylla Rafaella Sena Albuquerque, Nívea Maria Pacheco, Thalia del Rosario Loyo Casao, Fabiana Cristina Silveira Alves de Melo, Rômulo Dias Novaes, Reggiani Vilela Gonçalves

**Affiliations:** ^1^Department of Biochemistry and Biotechnology, Federal University of Viçosa, Viçosa, MG, Brazil; ^2^Department of Animal Biology, Federal University of Viçosa, Viçosa, MG, Brazil; ^3^Institute of Biomedical Sciences, Department of Structural Biology, Federal University of Alfenas, Alfenas, MG, Brazil

## Abstract

Melanoma is the most aggressive form of skin cancer and arises from melanocyte gene mutation. This disease is multifactorial, but its main cause is the excessive exposure to ultraviolet radiation. Currently, available chemotherapy has shown little expressive results, which may justify the high use of natural products to treat this cancer. We performed a systematic review to compile the results of studies carried out in murine models and investigated the effect of plant extracts on melanoma treatment. Papers were selected in MEDLINE/Pubmed and Scopus according to the PRISM statement. Search filters were developed using three parameters: plant extract, melanoma, and animal model. The 35 identified studies were all submitted to the criteria described in the ARRIVE guidelines. The different extracts showed antiangiogenic, antimetastatic, antioxidant, and anti-inflammatory activity, and also proved to be effective in cell cycle modulation and apoptosis evasion. Bias analysis evidenced the absence of standardized experimental designs, as well as failures in statistical tests and in the presentation of results. The analysis of the studies suggests that the use of plant extracts is effective for the treatment of melanoma in murine models.

## 1. Introduction

According to the World Health Organization (WHO), cancer is a prevalent public health problem that leads to high mortality rates worldwide. American data shows that skin cancer is the most common in the country [1, 2]. In Brazil, skin cancer accounts for 25% of all registered malignant tumors [3, 4]. Only 3 to 4% of cutaneous tumors are diagnosed as melanoma, though this represents the most severe form of the disease, mainly due to its high metastatic rates [2, 3, 5]. Cutaneous melanoma is the most common among the Caucasian population, while pigmented populations in Africa and Asia develop lesions on the soles of the feet and mucous membranes, both with low incidence rates [6]. Globally, melanoma is responsible for an average of 232,000 new cases per year, and high mortality rates occur in developing countries [2, 5].

Melanoma originates from the malignant transformation of melanocytes, which are melanin-producing cells from the neural crest, and the skin is its primary site [6, 7]. It has been shown that melanoma is the result of multiple and progressive DNA damage, which can be caused by protooncogene activation, mutations, and/or deletions of tumor suppressor genes, as well as structural changes of the chromosomes [5–9]. Genetic factors should also be considered a risk factor, since about 10% of melanoma cases are diagnosed in individuals with a family history of the disease [10].

Although malignant melanoma is considered a multifactorial disease, the main cause of its development is the excessive exposure to ultraviolet (UV) radiation [2, 5–7]. The main source of this type of radiation is sunlight exposure, which induces DNA damage, genetic mutations, and a rise in the inflammatory response. These factors contribute to the increased risk of developing various types of skin cancer [11, 12].

The major signal transduction pathways that are involved in the development of melanoma are the MAP kinases and phosphatidylinositol-3-kinase (PI3K). In general, hyperactivation of these pathways occurs through mutations and gene deletions [12]. Changes in the proteins involved in this process are usually related to an increase in the metastasis and, when undiagnosed and not treated early, the tumor tends to increase in depth and height with progressive alteration of the original tissue [7, 11].

Localized melanoma can be curable by surgical excision with adequate safety margins [9, 11]; however, metastatic melanoma brings a worse prognosis [5–7, 11]. Patients with advanced melanoma present a 1-year survival rate, and about 25% have a median survival time of 6 months [13]. Nowadays, there are many treatment strategies that aim at blocking the migratory and invasive capacity of malignant cells [11]. The cellular response to most chemotherapeutic agents is generally low and is associated with high cell resistance, mainly when diagnosed at an advanced stage [3, 5–7, 14]. In this context, the search for natural products for the prevention and treatment of cancer has grown considerably, since some molecules extracted from these compounds have shown anti-inflammatory, proapoptotic, and antiangiogenic effects [1, 11, 15]. In this review, the main compounds found were phenolic compounds, mainly flavonoids, phenolic acids, and tannins, followed by terpenes, alkaloids, curcumin, and saponins. However, the parameters used to evaluate the efficacy of natural products for melanoma treatment, as well as the results obtained, are heterogeneous, which makes the literature inconsistent. Some clinical and preclinical studies have tried to demonstrate the positive effect of plant compounds and their derivatives for the treatment of melanoma. However, these hypotheses are not always confirmed, mainly due to the methodological variations involving different extraction protocols, therapeutic schemes, and mechanisms of action. Therefore, it is crucial to compile data from various studies in order to clarify the mentioned discrepancies. In this context, the systematic review is a powerful tool that incorporates variability between studies, and obtains an evaluation of the use of plant extracts for the treatment of melanoma in murine models. This systematic review was developed to determine if there is a rational basis for the selection of all investigated plant species, taking into account the geographic distribution of each species as well as any evidence of ethnodirected bioprospecting. Furthermore, we performed a critical analysis of preclinical studies in order to improve the quality of the reports and prevent the reproduction of methodological failures, which could compromise the development of clinical studies.

## 2. Material and Methods

The systematic review adhered to PRISMA (Preferred Reporting Items for Systematic Reviews and Meta-Analysis) [16] guidelines, including search strategy, selection criteria, data extraction, and data analysis.

### 2.1. Search Strategy

We performed an extensive bibliography search using the electronic databases PubMed (https://www.ncbi.nlm.nih.gov/pubmed) and Scopus (https://www.scopus.com/home.uri), completed in May 10, 2018 at 4:00 p.m. The keywords used were based on filters constructed by three criteria: plant extract, melanoma, and animal model. The filters were developed from the PubMed database according to the hierarchical distribution of medical subject headings (MeSHTerms). The terms used to search on PubMed were adapted for the selection of Scopus publications, and the “animal model” filter was provided by the site itself [17] (Table S1 in Supplementary Material). Only experimental studies written in English or Portuguese were included. Reviews, comments, and notes, as well as unpublished studies, were not considered. The studies were selected based on the inclusion criteria described below:

- Studies investigating the effect of plant extracts for the treatment of melanoma were included.
- Studies in murine models were included.
- Studies testing isolated, synthetic, essential oils and fractions obtained from plant extracts were removed.
- *In vitro* studies of melanoma and other tumors, as well as studies reporting only the antimelanogenic effect, were excluded.
- Studies reporting the use of crude extracts for the treatment of other tumors and metastasis in organs other than the skin were also removed.

### 2.2. Data Extraction

An initial selection based on title and abstract (TIAB) was conducted by three independent reviewers (KRSA, NMP, and TRLC). In case of disagreements, a fourth reviewer (RDN) decided whether the study met the inclusion and exclusion criteria.

In order to discard subjectivity in the data collection and selection strategy, the information was independently extracted by the four reviewers (KRSA, NMP, TRLC, and RDN) and analyzed separately. Data from each study was extracted and tabulated using standardized information, such as publication characteristics (author, title, journal, year, and country), plants (plant species, used parts, and popular indication), research methods (control group, randomization, experimental procedures, and evaluation of the results), experimental model (animal, number of animals, sex, age, weight, species, acclimation period, experimental groups, food supply, temperature, and light cycle), melanoma induction (methods of induction and genetically modified species), melanoma monitoring (measurement size and range), and treatment description (routes of administration, treatment time, treatment response, and biochemical variations). When there was difficulty in extracting the data or obtaining the papers, the authors were contacted by email to provide the necessary information. Divergent opinions were resolved by consensus among reviewers.

### 2.3. Bias Analysis

The article quality was analyzed by the criteria described on the ARRIVE platform (Animal Research: Reporting of *In Vivo* Experiments). These criteria were based on short descriptions of essential characteristics of all studies with animal models such as theoretical and methodological basis, research objective, improvement of analytical methods, statistical design, sample calculations, and measured outcomes [18]. Considering the purpose of the systematic review on evaluating important aspects of the referenced publications, we built a table summarizing all the aspects investigated, as well as their relevance, that describes the characteristics of the recovered studies.

## 3. Results

### 3.1. Included Studies

**Figure 1:**
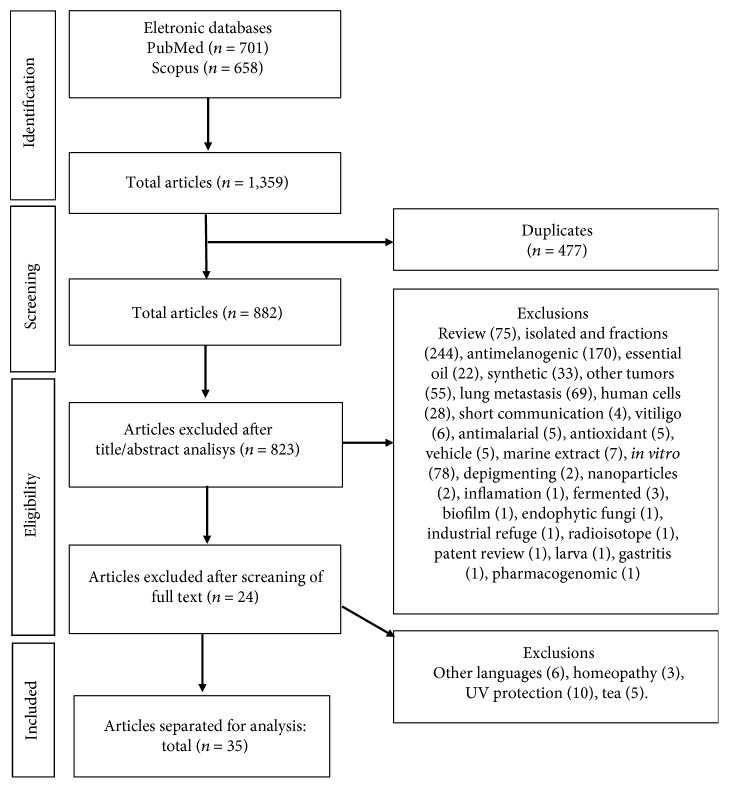
Flow diagram of the systematic review literature search results. Based on [16].

The PRISMA diagram illustrates the process of selecting studies (Figure 1). We found a total of 1359 papers, out of which 477 were duplicates. After reading the titles and abstracts, 823 studies were excluded due to an inadequate research topic. Among the excluded studies, we highlighted 244 studies focused on fractions and isolated plants, 75 consisting of nonoriginal research articles (reviews), 55 related to other types of tumors, 69 investigating only pulmonary metastasis, 170 evaluating only the antimelanogenic effect of plant extracts, 33 evaluating the efficacy of synthesized compounds, and 78 investigating the effect of plant extracts on *in vitro* melanoma. The 59 remaining articles were carefully analyzed, of which 24 were excluded because they did not meet the eligibility criteria. Next, 35 studies were selected and their reference lists were screened to identify additional relevant studies missed in the initial search strategy, and none of them were included.

### 3.2. Qualitative Analysis

**Figure 2:**
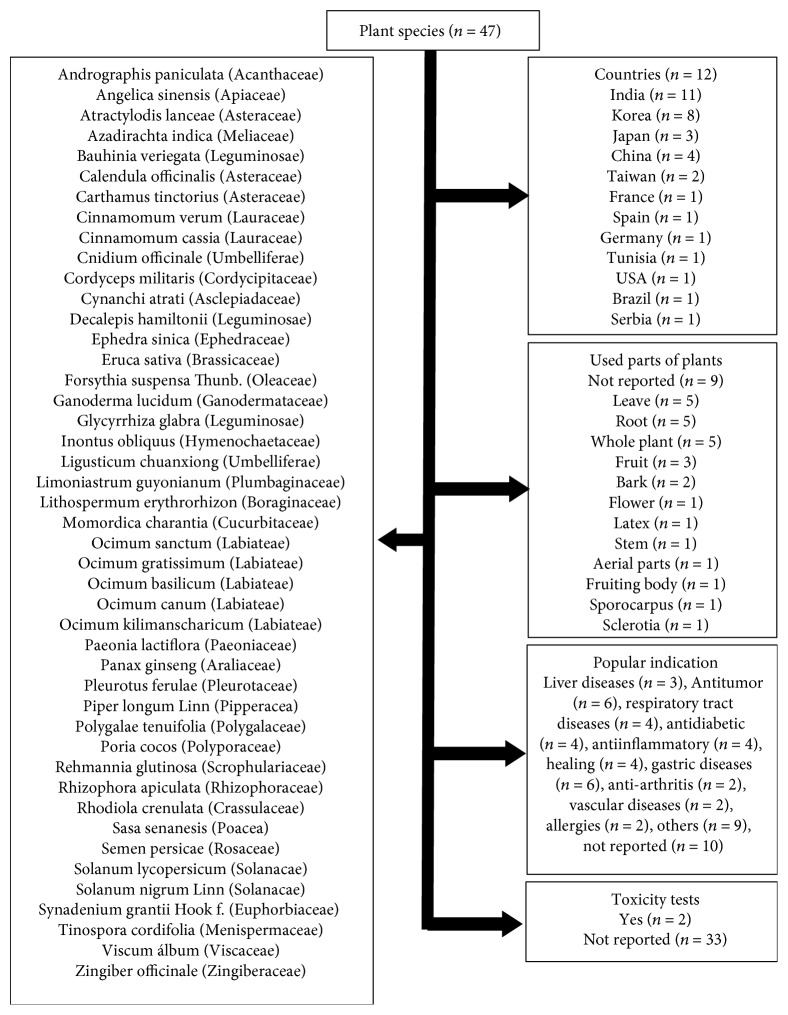
Summary of the studies describing the plant species, families, used parts of plants, toxicity tests, and popular indications.

The analyzed studies were conducted in 12 different countries, especially India (*n* = 11), followed by Korea (*n* = 8), China (*n* = 4), and Japan (*n* = 3). Forty-seven plant species were investigated and only 1 study did not report the species. The following families were included in the studies: Ephedraceae, Menispermaceae, Asteraceae, Pipperacea, Acanthaceae, Solanacae, Hymenochaeraceae, Cucurbitaceae, Lauraceae, Brassicaceae, Labiateae, Rhizophoraceae, Zingiberaceae, Leguminosae, Asclepiadaceae, and Euphorbiaceae. However, 16 authors did not report the plant family. The most used plant structures were roots (*n* = 5), leaves (*n* = 5), and the whole plant (*n* = 5), followed by fruits (*n* = 3) and barks (*n* = 2). Many of the studies (*n* = 9) did not mention this information. Only 2 studies reported toxicity tests. The main results for plant species, families, used parts of plants, toxicity tests, and popular indications are shown in Figure 2.

Most studies included only male animals (*n* = 15), 8 used females, 3 used both sexes, and 9 studies did not provide this information. As for animal strain, most studies used mice of the C57BL/6 strain (*n* = 18), followed by BALB/c (*n* = 5), C57BL (*n* = 3), transgenic/304/B6 (*n* = 2), C57BL/6J (*n* = 1), C57BL/6N (*n* = 1), Athimic nude (*n* = 1), BALB/cAnN-Foxn1nu/CrlNarl nude (*n* = 1), BALB/c nu/nu (*n* = 1), C57BL/6NCrL (*n* = 1), and BDF1 (*n* = 1). The animals' age ranged from 4 to 12 weeks (*n* = 32), and 3 studies did not report this information. The weight ranged from 18 to 25 g (*n* = 18), though 17 studies did not report this data. Melanoma induction occurred in 33 studies and was performed mainly by injection of the B16F10 melanoma cell line (*n* = 26), followed by the B16 melanoma cell line (*n* = 5), ANDO-2 human melanoma cell line (*n* = 1), and A375 human melanoma cell line (*n* = 1). Two studies used genetically modified organisms, not requiring, therefore, the injection of melanoma cells (*n* = 2).

There was a large variation in the amount of extracts administered in the animals, but in general, this was performed by oral administration (*n* = 17). Doses ranged from 50 *μ*L to 1000 mg/kg/body weight. Intraperitoneal injection was reported in 11 studies with doses ranging from 20 *μ*g to 400 mg/kg/body weight. Some studies used both posologies to evaluate the best method (*n* = 2). Only 1 study reported topical application of the extract directly on the tumor without mentioning periodicity. In other studies (*n* = 2), the extract was injected subcutaneously into the peritumoral region at intervals of two days. In 2 studies, neither frequency nor route of administration was reported.

The treatment time was divergent among the studies. Most studies covered periods ranging from 5 days to 18 months. However, 2 studies extended the treatment until animal death, and 2 studies established (as a parameter) a limiting volume of the primary tumor to determine the end of the experiment (Table 1).

### 3.3. Main Parameters Analyzed to Evaluate the Extract Action on Melanoma

The main parameter used to analyze plant-extract effect on the development of melanoma was the scaling of primary tumors (*n* = 32). From the studies investigated, 22 measured tumor volume, 7 measured tumor weight, and 2 measured tumor weight and volume. Periodicity of tumor volume measurement was informed in 23 works, with an average interval measurement of once daily and every two or three days. Most works (*n* = 19) explained the way rates of tumor inhibition were calculated. In 30 studies, significant reductions occurred in tumor weight and/or volume in the experimental groups undergoing therapeutic treatment with plant extracts. One study reported tumor volume reduction only with the prophylactic use of the extract rich in flavonoids and terpenes. Another study using extract rich in phenolic compounds reported having found no effect of the plant extract on tumor growth (Table 2).

Another parameter used to measure extract effectiveness was the emergence of metastasis (*n* = 6). This parameter was analyzed by counting the number of metastatic nodules (*n* = 3), and measuring spleen and lymph node weight (*n* = 1) and pulmonary weight (*n* = 1). Metastasis measurement protocol was not reported in 1 study. Significant metastasis reductions related to plant-extract treatment were reported in 5 studies, whose predominant compounds were flavonoids, saponins, essential oils, tannins, and phenolic compounds, and 1 study reported no histological differences between control and treated groups (Table 2).

The impact of plant extracts on animal survival was also analyzed (*n* = 15), and 14 studies reported that the survival rate increased after treatment with extracts. They also measured the tumor latency period, that is, the time the tumor takes to go from benign to malignant stage (*n* = 4). Only 1 study reported a significant increase of this parameter.

**Figure 3:**
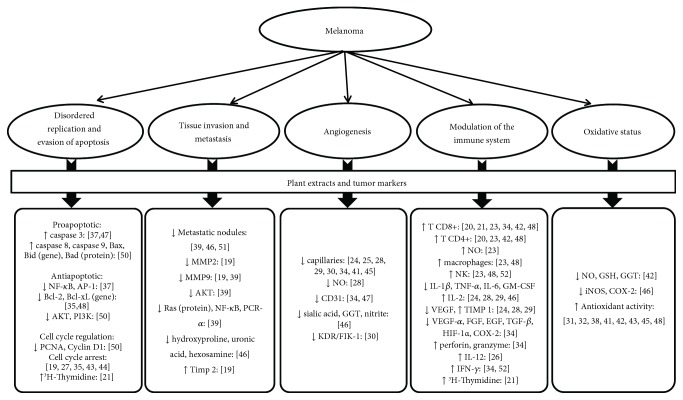
Article results. NF-*κ*B (nuclear factor kappa B), AP-1 (activator protein 1), AKT (protein kinase B), PI3K (phosphatidylinositol-3-kinase), PCNA (proliferating cell nuclear antigen), MMP-2 and MMP-9 (metalloproteinases 2 and 9), PKC*α* (protein kinase C alpha), TIMP-1 and TIMP-2 (metallopeptidase inhibitors 1 and 2), NO (nitric oxide), GGT (gama-glutamil transpeptidase), KDR/FIK-1 (kinase insert domain receptor/fetal liver kinase 1), NK (natural killers), IL (interleukin), IFN-*γ* (interferon gama), TNF-*α* (tumor necrosis factor alfa), GM-CSF (granulocit-monocit colony stimulating factor), VEGF (vascular endothelial growth factor), FGF (fibroblast growth factor), EGF (epidermal growth factor), TGF-*β* (transforming growth factor-beta), HIF-1*α* (hypoxia-inducible factor-1*α*), COX-2 (cyclooxygenase 2), GSH (glutathione), and iNOS (inducible nitric oxide synthase).

Apoptosis was investigated by the quantification of proapoptotic and antiapoptotic parameters. The proapoptotic markers analyzed were caspase 3, 8, and 9, Bax and Bid genes, and Bad protein. All studies evaluating apoptosis found a significant increase in proapoptotic markers in the groups treated with plant extracts. The antiapoptotic markers analyzed were NF-*κ*B (nuclear factor kappa B), AP-1 (activator protein 1), gene Bcl, AKT (protein kinase B), and PI3k (phosphatidylinositol-3-kinase). All studies reported a decrease in these markers after plant-extract treatment (Table 2 and Figure 3).

Cell replication was investigated through cell cycle markers such as PCNA (proliferating cell nuclear antigen), cyclin D, and ^3^H-Thymidine (Figure 3). The first two markers were reduced and the third was increased after plant-extract treatment. The main compounds present in these extracts were saponins, terpenes, and flavonoids (Table 2).

Tissue invasion and metastasis were explored by analyzing characteristic markers such as metalloproteinases (MMP), AKT, Ras protein, NF-*κ*B, PKC*α* (protein kinase C alpha), hydroxyproline, uronic acid, and hexosamine. All these markers showed a reduction after plant extract treatment containing predominantly flavonoids, saponins, and polyphenols. TIMP-2 (metallopeptidase inhibitor 2) was also investigated and showed an increase after the treatment with extract rich in flavonoids and saponins (Table 2).

Angiogenesis was histologically analyzed in 8 studies by peritumoral capillary counting, and a reduction in the number of vessels was observed after treatment with extracts containing predominantly alkaloids, terpenes, phenolic compounds, essential oils, tannins, flavonoids, and carotenoids (Table 2). This was also measured through serum levels of NO (nitric oxide), nitrite, CD31, sialic acid, GGT (gamma-glutamyl transpeptidase), and KDR/FIK-1 (kinase insert domain receptor/fetal liver kinase 1). All these markers presented reductions after a plant-extract treatment rich in terpenes, essential oils, tannins, saponins, flavonoids, and alkaloids (Table 2).

The effect of plant extracts on immune modulation was explored through parameters of the innate and acquired immune system. Plant-extract treatment containing predominantly saponins, flavonoids, terpenes, essential oils, tannins, anthraquinones, phenolic compounds, alkaloids, and lectins increased levels of the following markers: NO, macrophages activity, cytotoxic activity of NK (natural killers) cells, phagocytic activity, activation of splenic cells, T helper cells (CD4+), activation of cytotoxic T cells (CD8+), T helper cells (CD4+), NO, macrophages activity, cytotoxic activity of NK (natural killers) cells, IL-2 and IL-12, TIMP-1 (metallopeptidase inhibitor 1), perforin, granzyme, IFN-*γ* (interferon gamma), and ^3^H-thymidine. The levels of the following markers, on the other hand, were reduced by plant-extract treatment rich in alkaloids, terpenes, phenolic compounds, essential oils, tannins: IL-1*β*, TNF-*α* (tumor necrosis factor alpha), IL-6, GM-CSF (granulocit-monocit colony stimulating factor), VEGF (vascular endothelial growth factor), FGF (fibroblast growth factor), EGF (epidermal growth factor), TGF-*β* (transforming growth factor-beta), HIF-1*α* (hypoxia-inducible factor-1 *α*), and COX-2 (cyclooxygenase 2) (Table 2).

Tumor oxidative status was assessed by measuring serum levels of NO, GSH (glutathione), GGT (gamma glutamyltransferase), iNOS (inducible nitric oxide synthase), and COX-2. All these markers showed significant reductions after plant-extract treatment, which contained flavonoids, tannins, catechins, anthraquinones, phenolic compounds, and saponins (Table 2 and Figure 3).

### 3.4. Bias Analyses

From the works analyzed, 88.5% were consistent with the title content. However, only 51.4% showed abstracts containing literature review, objectives, methods, main results, and conclusions. Ethics committee approval was reported in 71.4% of the studies. The experimental design was demonstrated in 88.5% of the studies, but only 37.1% reported measures taken to minimize bias in animal studies. Regarding animal experimentation, most studies (94.3%) informed the dose and method of administration.

All studies reported animal species used for investigating the melanoma. Sex was reported in 71.4%, animal weight was described in 51.4% of the works, and 91.4% of them reported the animals' age. Description of statistical analysis was reported in 88.5% of the studies. Only 1 study reported experimental protocol modifications due to adverse events. No study reported choice criterion for the number of animals used (Table 3).

## 4. Discussion

### 4.1. General Aspects

In this work, we performed a systematic review to study the effect of plant extracts in the treatment of melanoma in murine models. Despite the great heterogeneity of the studies, we observed that, in general, plant extracts are effective in melanoma treatment. Our option for researches carried out with plants is due to the fact that they are the oldest and most important source of bioactive compounds for the treatment of various diseases, and they contribute directly to the development of new drugs in the advances of chemical synthesis [11, 54, 55]. In addition, there is a growing interest in the use of natural sources for the development and formulation of new products as an alternative to conventional medicines and synthetic products [56–58]. The use of plant extracts for the treatment of different tissue changes is an ancient practice, and it is the closest to popular use. It is worth mentioning that the biological activity of a natural product is often due to the synergy between its constituents, which enhances its therapeutic properties [59, 60]. Thus, therapy with natural products currently represents a promising alternative for cancer treatment, especially melanoma, which is classified as an extremely aggressive type of cancer, with high mortality worldwide. With this in mind, the present study is of great importance, as the discovery of new alternatives for the treatment of skin cancer brings hope to improve the patient's quality of life and reduce immediate costs.

In this review, the *in vivo* testing was chosen instead of *in vitro* due to the fact that tumor cells develop various strategies to evade the host's immune system [61]. While tumor cells are effectively detected and lysed by natural killer cells *in vitro*, they develop efficient mechanisms to evade the innate immune system *in vivo* [62]. As one of our goals was to find experimental models closest to the human one, we focused on the murine model, with tumor induction protocols suited for the murine lineage. Thirty-three studies induced tumors locally by subcutaneous, intradermal, intramuscular, or intraperitoneal injection of melanoma cell lineages. Six of these studies also conducted metastatic melanoma induction tests, and tumor inductions were performed through melanoma cell injection via the tail vein.

### 4.2. Main Parameters Analyzed and their Findings

Normal cells have different control mechanisms over the cell cycle, which are less efficient in tumor cells [63, 64]. In this review, cell cycle regulation was mainly analyzed by the histological examination of tumor tissues, in which larger percentages of cells in the G0/G1 phase were reported, characteristic of regulated mitotic activity. The works also analyzed the main tumor molecular markers, since it is known that cancer shares certain specific biological capabilities acquired during various stages of development [65, 66]. The main markers involved in tumor progression were organized into the following categories: disorderly replication and evasion of apoptosis, tissue invasion and metastasis formation, angiogenesis, modulation of the immune system, and oxidative status. It is important to note that all tumor metabolic pathways interrelate, so the same tumor marker may be involved in one or more pathways.

#### 4.2.1. Disorderly Replication and Evasion of Apoptosis

Tumor growth is related to an imbalance between proliferation and cell death. Disturbances in this system result in homeostasis breakage, with tissue increase or decrease [67]. Regarding disordered replication of tumors, 2 papers reported the effect of plant extracts rich in flavonoids, saponins, and terpenes in cell cycle arrest. This data corroborates literature findings; for example, Kaempferol, one of the most abundant flavonoids, has been found to block choroidal melanoma cell cycle progression in the G2/M phase [68]. Steroidal saponins also have been extensively studied for its antitumor effect. Several of these saponins inhibit tumor cell growth by cell cycle arrest and apoptosis [69]. Andrographolide, a diterpene present in *Andrographis paniculate*, inhibited the *in vitro* proliferation of different tumor cell lines, exercising direct anticancer activity on cancer cells by cell cycle arrest at the G0/G1 phase through the induction of the cell cycle inhibitory protein [70].

Programmed cell death, also known as apoptosis, strictly controls the pathway responsible for removing unwanted or damaged cells. Apoptosis is regulated by pro- and antiapoptotic signals that control sophisticated molecular mechanisms of induction or suppression of cell signaling cascades [71]. Tumor cells acquire the ability to escape from apoptotic signs [72, 73] and, on the other hand, show hyperactivity in response to promitotic signs [74]. These properties lead to uncontrolled cell division and decreased apoptosis, resulting in rapid tumor growth [73, 74]. We believe that an effective therapeutic agent for tumor growth control must be able to activate proapoptotic signaling pathways and/or inhibit antiapoptotic pathways in addition to being able to control cell division through cell cycle modulation. Our findings show that extracts rich in saponins, terpenes, and flavonoids exhibit the abovementioned activities through cell cycle modulation mechanisms. Literature shows, for instance, that a terpene found in *Ganoderma lucidum* extracts had shown strong antimelanoma activity *in vitro* and that it has reduced tumor volume *in vivo*, exercising these activities through oxidative stress, apoptosis induction, and the inhibition of cell proliferation [33]. Treatment with saikosaponin D, a saponin isolated from *Bupleurum falcatum*, inhibited cell proliferation by inducing apoptosis and blocking cell cycle progression in the G1 phase [75]. Eupatilin, a flavonoid isolated from the artemisia plant, induces apoptosis and G2/M phase cell cycle arrest in human melanoma A375 cells [76].

One of the central components in apoptosis and cell cycle regulation is NF-*κ*B, an important factor that induces the transcription of various antiapoptotic proteins and promotes cellular replication [77, 78]. Among the main apoptotic pathways, we can highlight the caspases, which are responsible for a series of processes that cleave the DNA and the cytoskeleton [79], alter the permeability of the mitochondrial membrane, and release cytochrome *c*, which is an effector molecule of the apoptotic processes [77, 78, 80]. Our studies have shown that, in general, plant extracts containing flavonoids, phenolic compounds, essential oils, tannins, terpenes, and saponins, activate proapoptotic pathways and reduce cell replication, which results in decreased tumor growth. These findings show the therapeutic potential of plant extracts on cancer treatment, since our results revealed that the extracts used promoted a significant reduction of tumor volumes. Literature shows that another common flavonoid, epigallocatechin-3-gallate (EGCG), is capable of inducing apoptosis and cell cycle arrest in melanoma cells. The mechanisms through which EGCG exerts these effects include the downregulation of apoptosis-inhibiting proteins and cell survival-promoting proteins, the upregulation of proapoptosis proteins, and the activation of caspases 3, 7, and 9 [81].

#### 4.2.2. Tissue Invasion and Metastasis

Tumor metastasis encompasses several interrelated phenomena that consists of invasion and metastatic colonization in which malignant cells spread from the primary tumor to the organs [24, 64, 72, 82]. The metabolic pathways of disorderly replication and apoptosis evasion are involved in the metastatic process, therefore, many markers are common to both, such as NF-*κ*B, Ras, AKT, and PKC [83, 84]. For tissue invasion to occur, it is necessary that the MMPs cleave the basal membrane and the extracellular matrix, allowing the migration of these cells to the bloodstream [85]. Twenty-five MMPs have been characterized, as well as 4 of its inhibitors (TIMPs) [86]. The expression of MMPs can be activated by cytokines and growth factors, including TGF-*α* and *β*, interleukins, TNF-*α*, interferons, FGF, and VEGF [87]. Together with its inhibitors, such as TIMP-1 and TIMP-2, the MMPs keep a balance between collagen production and degradation. Each of these proteinases have specific roles in determining tumor invasive capacity [86]. MMP-2 and MMP-9 are especially relevant in melanoma progression, since they play an important role in tumor development, growth, angiogenesis, and metastasis. Thus, MMPs and their regulators should be considered potential targets for the development of antineoplastic drugs or chemotherapeutic agents [85]. Other components of the extracellular matrix are also increased during metastasis formation, such as hydroxyproline, uronic acid, and hexosamine [88]. The result of our work showed that all these markers have been assessed and that the analysis of metalloproteinase was present in most studies. The markers assessed showed reductions after plant-extract treatment with a high concentration of flavonoids, saponins, and polyphenols, except for the metalloproteinases inhibitors, which increased after the use of these extracts. These findings show that extracts protect the extracellular matrix from degradation, making it difficult for tumor cells to migrate, slowing the invasion of capillaries and, consequently, lowering metastasis formation and invasion of other tissues. Literature shows that treatment of melanoma cells with curcumin, a plant rich in polyphenols, resulted in the inhibition of the NF-*κ*B prosurvival pathway and activation of the death receptor Fas-initiated Fas-associated protein with death domain (FADD) apoptotic pathway via caspase 8 [89]. To Man et al., soybean saponin inhibits tumor cell metastasis by suppressing the MMP-2 and MMP-9 productions, and stimulates TIMP-2 secretion [69].

#### 4.2.3. Angiogenesis

This process is a survival pathway of the tumor, which supplies the metabolic needs of the cells and acts as a route of excretion for tumor metabolism, and has a crucial role in tumor growth and metastasis generation [90]. Angiogenesis is regulated by a balance between pro- and antiangiogenic factors. During tumor progression, proangiogenic pathways are continuously activated, causing an uncontrolled growth of the peritumoral vascularity [91]. One of the key regulators of angiogenesis is VEGF, a potent mitotic agent of endothelial cells, which increases vascular permeability. Their levels are increased in inflammatory and neoplastic conditions and have been widely analyzed during tumor development [71, 90]. In this review, we investigated the activity of VEGF through one of its receivers (KDR/FIK-1) which is increased during angiogenesis [92]. Our findings showed that other markers, like CD 31, sialic acid, GGT, and nitric oxide, were also analyzed. This demonstrates the importance of these molecules on the formation of new blood vessels. These results are in agreement with other studies that proved that CD31 is an excellent marker of endothelial cells [93], that sialic acid and GGT are good markers of cell proliferation [94, 95], and that nitric oxide is an important vasodilator increasing the vascular permeability of tumors [96]. Our findings show that the use of plant extracts rich in terpenes, alkaloids, flavonoids, saponins, and other phenolic compounds inhibit angiogenesis and consequently decrease metastasis formation and tumor growth by inhibiting vascular growth markers such as VEGF and CD31. An active component of *Zingiber officinale*, (6)-gingerol, a phenolic compound, inhibits melanoma tumor growth by affecting the venous supply to the tumor, but it is capable of causing cell death through apoptosis [97]. Ginsenosides, a kind of saponin, inhibit tumor angiogenesis by suppressing its inducer in the endothelial cells of blood vessels, and prevent tumor cells from adhering, invading, and metastasizing [98]. Oxymatrine, an alkaloid compound extracted from the root of *Sophora flavescents*, exerts its antiangiogenic effect in cancer cells by targeting the NF-*κ*B pathway and hindering the activity of the vascular endothelial growth factor (VEGF) involved in stimulating vasculogenesis and angiogenesis [99].

#### 4.2.4. Modulation of the Immune System

Immune implications of melanoma were the most addressed subjects of the selected studies in this review. Tumors develop multiple strategies to escape the recognition mediated by immune cells, which selects cells resistant to immunological defenses [61]. One of the main consequences of this evasion of the immune system is the installation of an inflammatory microenvironment rich in inflammatory cytokines, growth factors, chemokines, among others, promoting growth, progression, angiogenesis, and metastatic spread of various types of cancer. These factors are produced by the tumor itself and the surrounding tissue and contribute decisively to the malignancy [100, 101]. Two signaling pathways connect cancer and inflammation. The intrinsic pathway is related to gene mutations, which causes the resulting cells to increase the production of proinflammatory mediators [73, 100]. The extrinsic pathway is triggered by environmental factors, such as UV radiation, and is related to the increase of inflammatory leukocytes and the release of mediators that stimulate cell proliferation [73, 100]. Both pathways result in the activation of transcription factors, among which NF-*κ*B and the hypoxia-inducible factor 1 (HIF-*α* 1) stand out. These transcription factors modulate the production of numerous inflammatory mediators such as growth factors (VEGF, FGF, EGF, and TGF-*β*), cytokines (TNF-*α*, GM-CSF, IL-6, IL-*β*, IL-2, and IL-12), and enzymes (COX-2, iNOS) [77, 102, 103].

The levels of the following proinflammatory markers were reduced by plant-extract treatment containing alkaloids, terpenes, phenolic compounds, essential oils, and tannins: IL-1*β*, TNF-*α*, IL-6, GM-CSF, VEGF, VEGF*α*, FGF, EGF, TGF-*β*, HIF-1*α*, and COX-2. On the other hand, the following mediators of the inflammatory response had their levels increased after treatment with plant-extracts rich in saponins, flavonoids, terpenes, essential oils, tannins, anthraquinones, phenolic compounds, alkaloids, and lectins: NO, activity of macrophages, cytotoxic activity of NK cells, phagocytic activity, activation of spleen cells, CD4+, CD8+, ^3^H-thymidine, Il-2, IL-12, TIMP-1, IFN-*γ*, granzyme, and perforin. Joint analysis of the data demonstrates that plant extracts are effective in decreasing proinflammatory markers and increasing anti-inflammatory markers, leading to a subsequent modulation of the inflammation associated to cancer. Some plant-derived compounds that show promising potential as immunomodulatory agents are alkaloids, phenolic compounds, glycosides, terpenes, polysaccharides, lactones, among others [104]. The extract of *Limoniastrum guyonianum*, rich in flavonoids, has immune-modulatory and antioxidant characteristics and might be utilized as a good, natural, immune-modulatory and antioxidant antitumoral agent [48]. The flavonol quercetin has been found to exert anti-inflammatory activities [104]. The terpene ginsan, from *Panax ginseng*, enhances the production of cytokines and stimulates the phagocytic activity of macrophages [104].

#### 4.2.5. Oxidative Stress

Oxidative stress is the imbalance of the cellular oxidative potential caused by the overproduction of reactive oxygen species (ROS) or by a decreased antioxidant potential. When it comes to regulating signal transduction pathways, ROS are involved in the generation of potential carcinogenics including self-sufficiency in growth signals, insensitivity to growth inhibitors' signals, apoptosis evasion, limitless replicative potential, tissue invasion and metastasis, altered metabolism, and inflammation [48]. The main markers of oxidative stress are by-products of membranes and proteins generated in the cell by the action of free radicals, as in NO, malondialdehyde, hydroperoxides, and carbonylated proteins, besides the action of antioxidant enzymes, such as GSH, GGT, iNOS, and COX-2 [105, 106]. The oxidative tumor status was assessed by the measurement of serum levels of NO, GSH, GGT, iNOS, and COX-2. All these parameters demonstrated significant reductions when treated with plant extracts containing flavonoids, tannins, catechins, anthraquinones, phenolic compounds, and saponins, showing the antioxidant capacity of this type of treatment. The main natural products with antioxidant properties are phenolic compounds (including flavonoids and tannins), and their radical scavenging activities are related to the substitution of hydroxyl groups in the aromatic rings of phenolics. Total phenolic content and total antioxidant activity in phytochemical extracts may have a direct relationship [107]. Flavonoids possess many biochemical properties, but the best-described property of almost every group of flavonoids is their capacity to act as antioxidants through several mechanisms of antioxidant activity such as radical scavenging and metal ion chelation ability [108]. The antioxidant activity of *Psychotria carthagenensis*, *P. leiocarpa*, *P. capillacea*, and *P. deflexa* (Rubiaceae) extracts, rich in total phenolics, flavonoids, tannins, and flavonols, was determined in different *in vitro* assays [109].

### 4.3. Limitations

Systematic review studies are characterized by high levels of evidence, since they allow the evaluation of a variability of individual works from multiple studies. Thus, a major contribution of this work is based on the global estimate of the use of plant extracts for melanoma treatment. However, the results presented herein must be interpreted with some caution, since the process of selection of papers may be skewed due to different factors such as initial deletion based on just reading the titles and abstracts or the inclusion of more than one study of the same group of researchers. However, paper selection was based on widely recommended and accepted practices for the conduct of the present review.

Another important issue highlighted in our work is related to publication bias. For this, we used the ARRIVE guide, which allowed us to test the bias of the publications individually and then collectively. After this analysis, we realized that aspects related to the organization of the experiments were neglected, including the lack of randomization. This point raised additional concerns, since randomization was reported in only 37.1% of the studies. These factors highlight the need to improve experimental designs and current guidelines on the report of animal experiments as a means to ensure an adequate level of scientific evidence. Finally, we observed that the methodologies and the parameters of evaluation were extremely heterogeneous among the studies, and all studies reported different measures, such as weight and volume analysis, as well as biochemical analyses. Curiously, most papers did not report whether the results of the studies could be extrapolated into other systems, and no relevance was made to human biology. Considering the experimental models used in most works and the social relevance of melanoma for the world population, the translation of the results and the applicability for human treatment is essential in order to allow the continuation of the studies on medicinal plants. The aim of this type of study is to develop a drug that improves patients' quality of life and brings us closer to finding a cure for skin cancer, the most common disease with a high mortality rate worldwide.

## 5. Conclusions

Plant extracts are effective in the treatment of melanoma, either by shrinking the tumor, reducing the metastatic potential, or assisting tumor angiogenesis regression. The repressing action on tumor responses happens through different metabolic pathways. As a consequence, the modulation of signaling carcinogenic pathways occurs, which decreases disorderly replication, apoptosis evasion, tissue invasion, metastasis formation, angiogenesis, and inflammation. However, the relevance of studies using plant extracts for melanoma treatment is hampered by the lack of methodological rigor. Thus, the need for the implementation of new methodologic protocols is imperative for experimental studies with animal models in order to ensure the repeatability, reliability, and generalizability of the results.

## Figures and Tables

**Table 1:** Description of the main characteristics of the studies using plant extracts for the treatment of melanoma in murine models.

Reference	Country	Plant species/family	Animal/strain	Sex	Age/weight	Number of groups	Number of animals per group	Melanoma induction	Control group	Dose	Administration/frequency	Tumor measurement/frequency	Tumor development calculation	Treatment time
Kato et al., 1998 [19]	Japan	*Bupleurum chinense*, *Pinellia ternata*, *Scutellaria baicalensis*, *Panax ginseng*, *Ziziphus jujuba*, *Glycyrrhiza glabra*, *Zingiber officinale* (Various botanical families)	Transgenic mice/304/B6	?	1 mo/?	2	90–94	Genome engineering	Water	3–5 mg/day/mouse	Oral/daily	Volume (mm^3^)/?	?	Until death of mice
Xiaoguang et al., 1998 [20]	China	*Panax ginseng* (Araliaceae)	Mice/C57/BL	♀	?/18–22 g	5	10	B16 cell suspension (0.2 mL) subcutaneously	CTX 100 mg/kg	50 mg/kg, 100 mg/kg, and 200 mg/kg	Oral/daily	Weight of tumor/end of experiment	Rates of inibition was calculated comparing the weight of tumors on treated and untreated groups	10 d
Dai et al., 2001 [21]	Japan	*Panax ginseng*, *Glycyrrhiza uralensis*, *Polygalae tenuifolia*, *Cinnamomum verum*, *Rehmannia glutinosa*, *Paeonia lactiflora*, *Cnidium officinale*, *Atractylodes lancea*, *Angelica sinensis*, *Poria cocos* (Various botanical families)	Transgenic mice/304/B6	?	1 mo/?	2	35	Genome engineering	Water	3–5 mg/day/mice	Oral/daily	Volume (mm^3^)/?	?	Until death of mice
Nam et al., 2003 [22]	Korea	*Ephedra sinica* (Ephedraceae)	Mice/BDF1	♂	?/18–20 g	4	8	B16F10 cells (1 × 10^7^ cells/mL) intradermally on the back	Saline and adriamycin	100, 30, and 10 mg/kg	8 injections/days 1, 2, and every 2 days following day 5	Volume (mm^3^)/days 1, 2, and every 2 days following day 5	Volume = width × length × height	18 d
Baral and Chattopadhyay, 2004 [23]	India	*Azadirachta indica* (Meliaceae)	Mice/C57/BL	♀	6 wk/25 g	?	?	B16 cells (1 × 10^6^) subcutaneously	PBS	0.5 mg/mouse 1x a wk	Injected/once a wk	Volume (mm^3^)/weekly	Volume (mm^3^) = (width^2^ × length)/2	4 wk
Leyon and Kuttan, 2004 [24]	India	*Tinospora cordifolia* (Menispermaceae)	Mice/C57BL/6	♂	4–6 wk/?	2	6	B16F10 (10^6^ cells/animal) intradermally on ventral skin	1% gum acacia in PBS	20 mg/kg	Intraperitoneal injection/5/doses at 24 h interval	?	?	9 d
Yoo et al., 2004 [25]	Korea	*Cordyceps militaris* (Clavicipitaceae)	Mice/C57BL/6	?	5-6 wk/?	3	?	B16F10 cells (5 × 10^5^) into foot pad	Distilled water	200 mg/L and 600 mg/L	?	Weight of removed foot pad	?	20 d
Duong Von Huyen et al., 2006 [26]	France	*Viscum album*(Viscaceae)	Mice/C57BL/6	?	8–10 wk/?	3	4–6	B16F10 cells (5 × 10^6^ cells/100 *μ*L in PBS) subcutaneously in the left flank	PBS	20 *μ*g/mice/day	Intraperitoneal injection/daily	Weight of tumor/end of experiment	?	7 d
Jiménez-Medina et al., 2006 [27]	Spain	*Calendula officinalis* (Asteraceae)	Mice/athimic nude	?	6 wk/?	6	10	Human melanoma cell ANDO-2 (5 × 10^6^) subcutaneously at the back foot pad	Saline and taxol 5 mg/kg	50 mg/kg of weight and 25 mg/kg of weight	Oral/3x/wk and intraperitoneal/2x/wk	Diameter tumor (mm)/3x a wk	?	9–12 wk
Sheeja et al., 2006 [28]	India	*Andrographis paniculata* (Acanthaceae)	Mice/C57BL/6	?	4–6 wk/20–25 g	3	?	B16F10 cells (10^6^) intradermally on T ventral surface on the shaven ventral surface	1% gum acacia	10 mg/dose/animal and 500 *μ*g/dose/animal	Intraperitoneal injection/?	?	?	5 d
Sunila and Kutan, 2006 [29]	India	*Piper longum* Linn (Pipperacea)	Mice/C57BL/6	♂	4–6 wk/20–25 g	3	6	B16F10 cells (10^6^) intradermally on ventral skin surface	PBS and TNP 470 (30 mg/kg)	10 mg/dose/animal	?	?	?	5 d
Xu et al., 2006 [30]	China	*Semen persicae*, *Carthamus tinctorius*, *Rehmannia glutinosa*, *Ligusticum chuanxiong*, *Paeonia lactiflora*, *Angelica sinensis* (Various botanical families)	Mice/C57BL/6J	♀	6–8 wk/18–20 g	6	10	B16 melanoma cells (6 × 10^6^/mL) subcutaneous vaccination in the right axilla	CTX and saline	2.5 g/kg, 5 g/kg, and 10 g/kg	Intragastrally/daily	Weight of tumor/end of experiment	Tumor-inhibiting rate = the average weight of the tumor in the saline group − the average weight of the tumor in the medication group/the average weight of the tumor in the saline group × 100%	21 d
Agrawal and Jain, 2009 [31]	India	*Solanum lycopersicum* (Solanacae)	Mice/C57BL/6	♂	6-7 wk/25 g	3	4	B16F10 (5 × 10^5^ cells/animal) subcutaneously	?	500 and 1000 mg/kg body weight	Oral/?	Volume/?	?	30 d
Agrawal and Pandey, 2009 [32]	India	*Bauhinia veriegata* (Leguminosae)	Mice/C57BL/6	♂/♀	6-7 wk/25 g	3	4	B16F10 cell (5 × 10^5^ cells/animal) subcutaneously	?	500 and 1000 mg/kg/body weight	Oral/?	Volume/?	?	30 d
Harhaji Trajković et al., 2009 [33]	Serbia	*Ganoderma lucidum* (Ganodermataceae)	Mice/C57BL/6	?	8–10 wk/20–25 g	3	10	B16 melanoma cells (2.5 × 10^5^) in the dorsal lumbosacral region	DMSO/PBS	100 mg/kg body weight	Intraperitoneal injection/daily	Volume (cm^3^)/every 2 to 3 days	Tumor volume (cm^3^) = (*π*/6) × tumor length × tumor width^2^	13 d
Kwon et al., 2009 [34]	Korea	*Cinnamomum cassia* (Lauraceae)	Mice/C57BL/6	♂	6–8 wk/?	3	?	B16F10 cells (1 × 10^6^) subcutaneously into the flanks	PBS	10 mg/dose (400 *μ*g/g mouse weight)	Oral and injection/?	Volume (mm^3^)/every 2 days	Tumor volume = width^2^ × length × 0.52	30 d
Youn et al., 2009 [35]	Korea	*Inonotus obliquus* (Hymenochaetaceae)	Mice/Balb/c	♂	6 wk/20-21 g	6	7–9	B16F10 (2 × 10^5^) intraperitoneally implanted	Saline	20 mg/kg and 200 mg/kg	Intraperitoneal injection or oral/daily	Weight of tumor/end of experiment	?	10 d
Agrawal and Beohar, 2010 [36]	India	*Momordica charantia* (Cucurbitaceae)	Mice/C57BL	♂/♀	6-7 wk/25 g	3	4	B16F10 cell (5 × 10^5^) injected	?	500 and 1000 mg/kg/body weight	Oral/daily	Volume (mm^3^)/?	?	30 d
Kwon et al., 2010 [37]	Korea	*Cinnamomum cassia* (Lauraceae)	Mice/C57BL/6	♂	6–8 wk/?	2	10	B16F10 cells (1 × 10^6^cell/0.1 mL) subcutaneously into the flanks	PBS	10 mg/dose (400 *μ*g/g mice weight)	Oral/?	Volume (mm^3^)/every 2 days	Tumor volume = width^2^ × length × 0.52	30 d
Seki and Maeda, 2010 [38]	Japan	*Sasa senanesis* (Poacea)	Mice/C57BL/6J	♂	6 wk/?	?	?	B16F10 cells (2 × 10^6^) subcutaneously implanted dorsally	?	0.1% of extract	Oral/daily	?	?	?
Wang et al., 2010 [39]	Taiwan	*Solanum nigrum* Linn (Solanaceae)	Mice/BALB/cAnN-Foxn1nu/CrlNarl nude	?	5 wk/?	3	5	B16F10 cells (1 × 10^6^) in 400 *μ*L of matrigel implanted into the flank	?	0, 0.5, and 1%	Oral/daily	Weight of tumor/end of experiment	?	14 d
Khoobchandani et al., 2011 [40]	India	*Eruca sativa* (Brassicaceae)	Mice/C57BL/6	?	6-7 wk/25 ± 5 g	7	5	B16F10 melanoma cells subcutaneously injected in foot pad	Doxorubicin and saline	Aerial methanolic extract and root methanolic extract (400 mg/kg/BW day)	Intraperitoneal injection/1st, 5th, 9th, and 13th day of treatment	Foot pad diameter (mm^3^)/daily	Tumor volume (mm^3^) = 4/3 × *π* × ((1/2 × smaller diameter)^2^ × (1/2 × larger diameter))	21 d
Monga et al., 2011 [41]	India	*Ocimum sanctum*, *Ocimum gratissimum*, *Ocimum basilicum*, *Ocimum canum*, *Ocimum kilimanscharicum* (Labiateae)	Mice/C57BL	♂	6–8 wk/24 ± 2 g	6	10	B16F10 cells (2 × 10^5^/mL) intradermally on vertical side	Olive oil	200 mg/kg/body weight of 50% extract in 50 *μ*L olive oil	Oral/daily	Volume (mm^3^)/every alternative days	Tumor volume (TV) = 0.4(*ab* ^2^). The length is *a*, and *b* is the breadth of the tumor implant (in mm). TVs were then converted into relative tumor volume (RTV) = (TVx)/(TVo), where TVo is the tumor volume at day 1 and TVx is the tumor volume on the following days	20 d
Prabhu and Guruvayoorappan, 2012 [42]	India	*Rhizophora apiculata* (Rhizophoraceae)	Mice/BALB/c	♂	4–6 wk/?	2	6	B16F10 melanoma cells (1 × 10^6^) intramuscular into the right hindlimb	?	10 mg/kg/body weight	Intraperitoneal injection/daily	Volume (mm^3^)/3 days interval for 1 mo	Tumor volume = (4/3)*πr*_1_^2^ *r*_2_, where *r*_1_ and *r*_2_ represent the major and minor diameters	10 d
Rajasekar et al., 2012 [43]	Korea	*Lithospermum erythrorhizon* (Boraginaceae)	Mice/C57BL/6	♀	6-7 wk/?	4	?	B16F10 cells (1 × 10^6^) subcutaneously in the right flank region	CTX in PBS	10 mg/kg and 0.1 mg/kg	Intraperitoneal injection/3 days intervals	Volume (mm^3^)/every 3 days and tumor weight	Tumor volume = *AB* ^2^/2, where *A* is the length and *B* is the width. Inhibition ratio (%) = ((*A* − *B*)/*A*) × 100, where *A* is the average tumor weight of the negative control and *B* is the average tumor weight of the treated group	21 d
De Oliveira et al., 2013 [44]	Brazil	*Synadenium grantii* Hook f. (Euphorbiaceae)	Mice/C57BL/6	♂	9 wk/20 g	2	?	B16F10 cells 5 × 10^4^ subcutaneously in the dorsum	?	50 *μ*L	Oral/3x daily	Volume (mm^3^)/?	Volume tumor = longitudinal (head-tail) × transverse (paw − paw) × 3/4p	7 d
Lee et al., 2013 [45]	Taiwan	*Zingiber officinale* (Zingiberaceae)	Mice/BALB/c nu/nu	♀	4-5 wk/?	2	5	A375 cells (1 × 10^7^) subcutaneously	Vehicle?	300 mg/kg	Subcutaneous injection/4x a week	Volume (mm^3^)/4 days intervals	Tumor volume = length × width^2^/2	35 d
Shathish et al., 2013 [46]	India	*Decalepis hamiltonii* (Leguminosae)	Mice/C57BL/6	♂	?/20–25 g	2	6	B16F10 melanoma cells (1 × 10^6^) intramuscular in the right limb	Vehicle gum acacia	20 mg/kg body weight	Intraperitoneal injection/daily	Volume (mm^3^)/5 days for 1 month	Tumor volume = (4/3)*πr*_1_^2^ *r*_2_, where *r*_1_ and *r*_2_ represent the major and minor diameters	10 d
Strüh et al., 2013 [47]	Germany	*Viscum album* (Viscaceae)	Mice/C57BL/6NCrL	♂	8–10 wk/?	3	8-9	B16F10 cells (1 × 10^6^) injected into slanks	2-Hydroxypropyl-*β*-cyclodextrin	12 *μ*g/kg	Subcutaneous peritumoral injections/10 cycles	Volume (mm^3^) (vertical and lateral)/every second day, starting at day 3	Tumor sizes were determined by the means of two measurements (vertical and lateral)	20 d
Krifa et al., 2014 [48]	Tunisia	*Limoniastrum guyonianum* (Plumbaginaceae)	Mice/Balb/C	♂	6–8 wk/18–22 g	4	30	B16F10 tumor cells (2 × 10^6^) subcutaneously into the right hind leg	PBS	25 and 50 mg/kg/body weight	Intraperitoneal injection/once every two days	Tumor weight/days 7, 14, and 21	Inhibitory rate (%) = ((*C* − Trt)/*C*) × 100%, where *C* is the average tumor weight in the tumor control mice and Trt is the weight in mice that had received extract	21 d
Son et al., 2014 [49]	Korea	*Cynanchum atratum* (Asclepiadaceae)	Mice/C57BL/6N	♂	12 wk/24–26 g	3	20	B16F10 cells (1 × 10^7^ in saline) subcutaneously into the right lower flanks	Distilled water	100 and 200 mg/kg	Oral/daily	Volume (mm^3^)/every 2 days	Tumor volume = (*A* × *B* ^2^)/2, where *A* is the larger, and *B* is the smaller of the two dimensions	10 d
Wang et al., 2014 [50]	China	*Pleurotus ferulae* (Pleurotaceae)	Mice/C57BL/6	♀	6 wk/?	3	10	B16F10 cells (2 × 10^5^) subcutaneously injected	Cisplatin and PBS	100 mg/kg	Oral/daily	Volume (mm^3^)/every 3 days and tumor weight/end of experiment	?	12 d
Dudek et al., 2015 [51]	USA	*Rhodiola crenulata* (Crassulaceae)	Mice/C57BL/6	♀	8 wk/?	3	10	B16F10 cells (1 × 10^6^) subcutaneously above the scapular foot pad	DMSO cream	5% *R. crenulata* cream, 10% *R. crenulata* cream	Topical/daily	Volume (mm^3^)/daily	Tumor volume = (tumor length × tumor width^2^)/2	Until tumor exceded 1500 mm^3^
Jang et al., 2015 [52]	Korea	*Panax ginseng* (Araliaceae)	Mice/C57BL/6	♂	7–8 wk/?	2	?	B16 melanoma cells (1 × 10^5^) subcutaneously into the foot pads of the right hindlimb	PBS	200 *μ*L/mice	Intraperitoneal injection/daily	Volume (mm^3^)/every other day for 2 wk	Tumor volume = tumor thickness × maximum tumor diameter × tumor perpendicular length	10 d
Bao et al., 2017 [53]	China	*Forsythia suspensa* Thunb. (Oleaceae)	Mice/C57BL/6	♀	10 wk/22 ± 2 g	2	8	B16F10 cells 5 × 10^4^ subcutaneously	Water	10 g/kg	Oral/day 0 and every 2 days	Volume (mm^3^)/?	?	Until tumor exceded 2500 mm^3^

♂: male; ♀: female; wk: week; d: days; CTX: cyclophosphamide; PBS: phosphate buffered saline; DMSO: dimethyl sulfoxide; ?: not reported.

**Table 2:** Secondary metabolites, global effects, and ethnodirected indication for malignant tumors of the studies using plant extracts for the treatment of melanoma in murine models.

Author/year	Plant species	Native/exotic	Main secondary metabolites	Global effects	Ethnodirected indication for malignant tumors
Kato et al., 1998 [19]	*Bupleurum chinense*, *Pinellia ternata*, *Scutellaria baicalensis*, *Panax ginseng*, *Ziziphus jujuba*, *Glycyrrhiza glabra*, and *Zingiber officinale*	Native	Glycyrrhizin and ginsenoside (saponins), liquiritin (flavonoid), xanthones, terpenes, paeoniflorin, polysaccharides, and ferulic acid	Modulation of the immune system	Yes
Xiaoguang et al., 1998 [20]	*Panax ginseng*	Native	Baicalin and baicalein (flavonoids), glycyrrhizin, saikosaponin, and ginsenoside (saponins)	↓ disordered replication Proapoptotic activity ↓ tissue invasion and metastasis	Yes
Dai et al., 2001 [21]	*Panax ginseng*, *Glycyrrhiza uralensis*, *Polygalae tenuifolia*, *Cinnamomum verum*, *Rehmannia glutinosa*, *Paeonia lactiflora*, *Cnidium officinale*, *Atractylodes lancea*, *Angelica sinensis*, and *Poria cocos*	Native	Ginsenoside (saponins) and polysaccharides	Modulation of the immune system	Yes
Nam et al., 2003 [22]	*Ephedra sinica*	Native	Flavonoids and limonoids (terpenes)	Modulation of the immune system	Yes
Baral and Chattopadhyay, 2004 [23]	*Azadirachta indica*	Native	Ephedrine (alkaloid)	Antiangiogenic and antiinvasive activities	Yes
Leyon and Kuttan, 2004 [24]	*Tinospora cordifolia*	Native	Alkaloids, glycosides, steroids, polysaccharides, terpenes, and phenolic compounds	Antiangiogenic activity Modulation of the immune system	Yes
Yoo et al., 2004 [25]	*Cordyceps militaris*	Native	Nucleosides, polysaccharides, sterols, proteins, amino acids, and polypeptides	Antiangiogenic activity	Yes
Duong Von Huyen et al., 2006 [26]	*Viscum album*	Native	Lectins, phytotoxins, phenolic acids, and flavonoids	Modulation of the immune system	Yes
Jiménez-Medina et al., 2006 [27]	*Calendula officinalis*	Native	Flavonoids, carotenoids, triterpenes, and saponins	Cell cycle arrest Proapoptotic activity	No
Sheeja et al., 2006 [28]	*Andrographis paniculata*	Native	Polyphenols, alkaloids, piperine, and rutin	Antiangiogenic activity Modulation of the immune system	Yes
Sunila and Kutan, 2006 [29]	*Piper longum* Linn	Native	Flavonoids, terpenes (andrographolides), and phenylpropanoids	Antiangiogenic activity Modulation of the immune system	Yes
Xu et al., 2006 [30]	*Semen persicae*, *Carthamus tinctorius*, *Rehmannia glutinosa*, *Ligusticum chuanxiong*, *Paeonia lactiflora*, and *Angelica sinensis*	Native	Licopen (carotenoid), flavonoids, and terpenes	Antioxidant activity	No
Agrawal et al., 2009 [31]	*Solanum lycopersicum*	Exotic	Flavonoids, fatty acids, and lectins	Antioxidant activity	No
Agrawal and Pandey, 2009 [32]	*Bauhinia veriegata*	Native	Melanine pigments, salts of metal ions and low-weight compounds, phenols, and betulin	↓ disordered replication Proapoptotic activity	No
Harhaji Trajković et al., 2009 [33]	*Ganoderma lucidum*	Native	Essential oils (cinnamic aldehyde and cinnamyl aldehyde), coumarins, and tannins	Antiangiogenic and antiinvasive activities Modulation of the immune system	Yes
Kwon et al., 2009 [34]	*Cinnamomum cassia*	Native	Charantin, pectin, glycosides, saponins, alkaloids, reducing sugars, resins, phenolic compounds, fixed oil, and free acids	Proapoptotic activity	Yes
Youn et al., 2009 [35]	*Inonotus obliquus*	Native	Essential oils (cinnamic aldehyde and cinnamyl aldehyde) and tannins	Proapoptotic activity	Yes
Agrawal and Beohar, 2010 [36]	*Momordica charantia*	Native	*β*-Glucan, lignins, and phenolics	Antioxidant activity	Yes
Kwon et al., 2010 [37]	*Cinnamomum cassia*	Native	Polyphenols, luteolin, apigenin, kaempferol, and coumarins	↓ tissue invasion and metastasis	Yes
Seki and Maeda, 2010 [38]	*Sasa senanesis*	Native	Flavonoids, sterols, and isothiocyanates	Antiangiogenic activity	Yes
Wang, et al., 2010 [39]	*Solanum nigrum* Linn	Native	Saponins, flavonoids, terpenes, and tannins	Antioxidant activity	Yes
Khoobchandani et al., 2011 [40]	*Eruca sativa*	Native	4-Pyrrolidinyl, pyrazole, ketone derivatives and thiazolidine-diones, flavonoids, tannins, catechin, anthroquinone, and phenolic groups	Modulation of the immune system Antioxidant activity	No
Monga et al., 2011 [41]	*Ocimum sanctum*, *Ocimum gratissimum*, *Ocimum basilicum*, *Ocimum canum*, and *Ocimum kilimanscharicum*	Native	Shikonin derivatives (naphtoquinones)	Cell cycle arrest Proapoptotic activity	Yes
Prabhu and Guruvayoorappan, 2012 [42]	*Rhizophora apiculata*	Native	Polyphenols, vitamin C, *β* carotene, flavonoids, and tannins, (6)-gingerol	Proapoptotic activity Antioxidant activity	No
Rajasekar et al., 2012 [43]	*Lithospermum erythrorhizon*	Native	Saponins, vanillin, lupeol, and pectic polysaccharides	↓ tissue invasion and metastasis Modulation of the immune system Antioxidant activity	Yes
De Oliveira et al., 2013 [44]	*Synadenium grantii* Hook F.	Native	Visco lectins (terpenes and oleanolic acid)	Proapoptotic activity Antiangiogenic activity	Yes
Lee et al., 2013 [45]	*Zingiber officinale*	Native	Terpenes and polysaccharides	↓ disordered replication Proapoptotic activity Antioxidant activity	Yes
Shathish and Guruvayoorappan, 2013 [46]	*Decalepis hamiltonii*	Native	Tannins, polyphenols, and flavonoids (epigallocatechin gallate)	Modulation of the immune system Antioxidant activity	No
Strüh et al., 2013 [47]	*Viscum album*	Native	Sibiricoses and steroidal glycosides	Proapoptotic activity	Yes
Krifa et al., 2014 [48]	*Limoniastrum guyonianum*	Native	Terpenoids, *β*-glucan, peptides, polysaccharides, organic acids, terpenes, mevinol, saponins, and steroids	Proapoptotic activity Cell cycle arrest	No
Son et al., 2014 [49]	*Cynanchum atratum*	Native	Phenolic compounds, pyrogallol, gallic acid, and *β*-sitosterol	↓ disordered replication ↓ tissue invasion and metastasis Modulation of the immune system	Yes
Wang et al., 2014 [50]	*Pleurotus ferulae*	Native	Ginsenosides (saponins) and polysaccharides	Modulation of the immune system	Yes
Dudek et al., 2015 [51]	*Rhodiola crenulata*	Native	Quinochalones, flavonoids, alkaloids, polyacetylene, aromatic glucosides, organic acids, paeoniflorin (terpen), amygdalin, polysaccharides, Z-ligustilide, and ferulic acid	Antiangiogenic activity	No
Jang et al., 2015 [52]	*Panax ginseng*	Native	Terpenes, steroids, cumarins, tannins, anthraquinones, and saponins	Proapoptotic activity Cell cycle arrest Modulation of the immune system	Yes
Bao et al., 2017 [53]	*Forsythia suspensa* Thunb.	Native	Pholyphenols (forsythosides) and pinoresinol (lignan)	Modulation of the immune system	No

**(a):** 

	Kato et al., 1998 [19]	Xiaoguang et al., 1998 [20]	Dai et al., 2001 [21]	Nam et al., 2003 [22]	Baral and Chattopadhyay, 2004 [23]	Leyon and Kuttan, 2004 [24]	Yoo et al., 2004 [25]	Duong Von Huyen et al., 2006 [26]	Jiménez-Medina et al., 2006 [27]	Sheeja et al., 2006 [28]	Sunila and Kutan, 2006 [29]	Xu et al., 2006 [30]	Agrawal and Jain, 2009 [31]	Agrawal and Pandey, 2009 [32]	Harhaji Trajković et al., 2009 [33]	Kwon et al., 2009 [34]	Youn et al., 2009 [35]	Agrawal and Beohar, 2010 [36]
Title																		
Accurate and concise description of the content of the article	✓		✓	✓	✓	✓	✓	✓	✓	✓	✓	✓	✓	✓	✓		✓	✓
Abstract																		
Summary of the background, objectives, methods, principal findings, and conclusions	✓		✓						✓	✓				✓	✓	✓		
Introduction																		
Background	✓		✓	✓	✓	✓	✓		✓	✓	✓	✓	✓	✓	✓	✓	✓	✓
Objectives	✓	✓	✓	✓	✓	✓	✓	✓	✓	✓	✓	✓	✓	✓	✓	✓	✓	✓
Materials and methods																		
*Ethical statement*																		
Indicate the nature of the ethical review permissions and relevant licenses			✓			✓		✓	✓	✓	✓		✓	✓	✓	✓	✓	✓
*Study design*																		
Number of experimental and control groups	✓	✓	✓	✓	✓	✓	✓	✓	✓	✓	✓	✓	✓	✓	✓	✓	✓	✓
Any steps taken to minimize the effects of subjective bias when allocating animals to treatment	✓		✓						✓			✓		✓	✓	✓		✓
The experimental unit	✓	✓	✓	✓	✓	✓	✓	✓	✓	✓	✓	✓	✓	✓	✓	✓	✓	✓
*Experimental procedures*																		
Doses	✓	✓	✓	✓	✓	✓	✓		✓	✓	✓	✓	✓	✓	✓	✓	✓	✓
Method of administration	✓	✓	✓	✓	✓	✓		✓	✓	✓		✓	✓	✓	✓	✓	✓	✓
Choose of dose	✓		✓							✓								
*Experimental animals*																		
Origin of animal	✓	✓	✓	✓	✓	✓	✓	✓	✓	✓	✓	✓	✓	✓	✓	✓	✓	✓
Species	✓	✓	✓	✓	✓	✓	✓	✓	✓	✓	✓	✓	✓	✓	✓	✓	✓	✓
Sex		✓		✓		✓					✓	✓	✓	✓		✓	✓	✓
Developmental stage	✓		✓		✓	✓	✓	✓	✓	✓	✓	✓	✓	✓	✓	✓	✓	✓
Weight		✓		✓					✓	✓	✓	✓	✓	✓	✓	✓		✓
*Housing and husbandry*																		
Housing				✓	✓	✓			✓		✓		✓	✓		✓	✓	
Husbandry conditions				✓	✓	✓	✓	✓	✓	✓	✓		✓	✓	✓	✓		✓
*Sample size*																		
Specify the total number of animals used in each experiment	✓	✓	✓	✓		✓	✓	✓	✓		✓	✓	✓	✓	✓	✓		✓
Explain how the number of animals was decided																		
*Allocating animals to experimental groups*																		
How animals were allocated to experimental group (AZAR)	✓		✓						✓			✓		✓	✓	✓		✓
*Experimental outcomes*																		
Clearly define the primary and secondary experimental outcomes assessed	✓	✓	✓	✓		✓	✓	✓	✓	✓	✓	✓		✓	✓	✓	✓	
*Statistical methods*																		
Provide details of the statistical methods used for each analysis	✓		✓			✓	✓	✓		✓	✓	✓	✓	✓	✓	✓	✓	✓
Specify the unit of analysis for each dataset	✓		✓		✓	✓	✓	✓		✓	✓	✓			✓	✓	✓	✓
Describe any methods used to assess whether the data met the assumptions of the statistical approach					✓			✓		✓								
Results																		
*Baseline data*																		
For each experimental group, report relevant characteristics and health status of animals before treatment or testing														✓				
*Numbers analyzed*																		
Report the number of animals in each group included in each analysis	✓	✓	✓	✓				✓	✓		✓				✓			✓
If any animals or data were not included in the analysis, explain why	✓	✓																
*Outcomes and estimation*																		
Report the results for each analysis carried out	✓	✓	✓	✓	✓	✓	✓	✓	✓	✓	✓	✓			✓	✓	✓	✓
*Adverse events*																		
Give details of all important adverse events in each experimental group			✓	✓											✓			
Describe any modifications to the experimental protocols made to reduce adverse events				✓														
Discussion																		
*Interpretation/scientific implications*																		
Interpret the results, taking into account the study objectives and hypotheses, current theory, and other relevant studies in the literature	✓		✓	✓	✓	✓	✓	✓	✓	✓	✓	✓			✓	✓	✓	
Comment on the study limitations including any potential sources of bias				✓													✓	
*Generalizability/translation*																		
Comment on whether, and how, the findings of this study are likely to translate to other species or systems, including any relevance to human biology															✓			
*Funding*																		
List all funding sources	✓		✓	✓				✓	✓					✓	✓	✓	✓	✓
Results (%)	65.7	40.0	68.6	62.9	51.4	57.1	45.7	54.3	65.7	57.1	60.0	57.1	51.4	65.7	74.3	68.6	57.1	62.9

**(b):** 

	Kwon et al., 2010 [37]	Seki and Maeda, 2010 [38]	Wang, et al., 2010 [39]	Khoobchandani et al., 2011 [40]	Monga et al., 2011 [41]	Prabhu and Guruvayoorappan, 2012 [42]	Rajasekar et al., 2012 [43]	De Oliveira et al., 2013 [44]	Lee et al., 2013 [45]	Shathish and Guruvayoorappan, 2013 [46]	Strüh et al., 2013 [47]	Krifa et al., 2014 [48]	Son et al., 2014 [49]	Wang et al., 2014 [50]	Dudek et al., 2015 [51]	Jang et al., 2015 [52]	Bao et al., 2017 [53]	% of items reported
Title																		
Accurate and concise description of the content of the article	✓	✓	✓	✓	✓	✓	✓	✓			✓	✓	✓	✓	✓	✓	✓	88.5
Abstract																		
Summary of the background, objectives, methods, principal findings, and conclusions	✓	✓	✓		✓		✓	✓			✓		✓	✓	✓		✓	51,4
Introduction																		
Background	✓	✓	✓	✓	✓	✓	✓	✓	✓	✓	✓	✓	✓	✓	✓	✓	✓	94.3
Objectives	✓	✓	✓	✓	✓	✓	✓	✓	✓	✓	✓	✓	✓	✓	✓	✓	✓	100.0
Materials and methods																		
*Ethical statement*																		
Indicate the nature of the ethical review permissions and relevant licenses	✓			✓	✓	✓	✓	✓	✓	✓	✓	✓		✓		✓	✓	71,4
*Study design*																		
Number of experimental and control groups	✓		✓	✓	✓	✓		✓	✓	✓		✓	✓	✓	✓		✓	88.5
Any steps taken to minimize the effects of subjective bias when allocating animals to treatment			✓	✓	✓				✓					✓				37,1
The experimental unit	✓		✓	✓	✓	✓	✓	✓	✓	✓	✓	✓	✓	✓	✓	✓	✓	97.1
*Experimental procedures*																		
Doses	✓		✓	✓	✓	✓	✓	✓	✓	✓	✓	✓	✓	✓	✓	✓	✓	94.3
Method of administration	✓	✓	✓	✓	✓	✓	✓	✓	✓	✓	✓	✓	✓	✓	✓	✓	✓	94.3
Choose of dose						✓		✓				✓						17.1
*Experimental animals*																		
Origin of animal	✓	✓	✓	✓	✓	✓	✓	✓	✓	✓	✓	✓	✓	✓		✓	✓	97.1
Species	✓	✓	✓	✓	✓	✓	✓	✓	✓	✓	✓	✓	✓	✓	✓	✓	✓	100.0
Sex	✓	✓			✓	✓	✓	✓	✓	✓	✓	✓	✓	✓	✓	✓	✓	71.4
Developmental stage	✓	✓	✓	✓	✓	✓	✓	✓	✓		✓	✓	✓	✓	✓	✓	✓	91.4
Weight				✓	✓			✓		✓		✓	✓				✓	51.4
*Housing and husbandry*																		
Housing		✓	✓	✓	✓	✓	✓		✓	✓		✓	✓	✓	✓		✓	62.8
Husbandry conditions	✓	✓	✓	✓	✓	✓	✓		✓	✓		✓	✓	✓	✓	✓	✓	80.0
*Sample size*																		
Specify the total number of animals used in each experiment	✓		✓	✓	✓	✓			✓	✓	✓	✓	✓	✓	✓		✓	80.0
Explain how the number of animals was decided																		0.0
*Allocating animals to experimental groups*																		
How animals were allocated to experimental group (AZAR)			✓	✓	✓				✓					✓				37.1
*Experimental outcomes*																		
Clearly define the primary and secondary experimental outcomes assessed	✓	✓	✓	✓	✓	✓	✓	✓	✓	✓		✓	✓	✓	✓	✓	✓	88.5
*Statistical methods*																		
Provide details of the statistical methods used for each analysis	✓	✓	✓	✓	✓	✓	✓	✓	✓	✓	✓	✓	✓	✓	✓	✓	✓	88.5
Specify the unit of analysis for each dataset	✓	✓	✓	✓	✓	✓	✓	✓	✓	✓	✓	✓	✓	✓	✓	✓	✓	85.7
Describe any methods used to assess whether the data met the assumptions of the statistical approach				✓	✓	✓				✓		✓	✓		✓	✓		31.4
Results																		
*Baseline data*																		
For each experimental group, report relevant characteristics and health status of animals before treatment or testing															✓			5.7
*Numbers analyzed*																		
Report the number of animals in each group included in each analysis	✓								✓	✓		✓	✓	✓			✓	45.7
If any animals or data were not included in the analysis, explain why											✓		✓					11.4
*Outcomes and estimation*																		
Report the results for each analysis carried out	✓		✓	✓	✓	✓	✓	✓	✓	✓	✓	✓	✓	✓	✓	✓	✓	91.4
*Adverse events*																		
Give details of all important adverse events in each experimental group											✓		✓		✓			17.1
Describe any modifications to the experimental protocols made to reduce adverse events																		2.8
Discussion																		
*Interpretation/scientific implications*																		
Interpret the results, taking into account the study objectives and hypotheses, current theory, and other relevant studies in the literature	✓		✓	✓	✓	✓	✓	✓		✓	✓	✓	✓	✓	✓	✓	✓	82.8
Comment on the study limitations including any potential sources of bias		✓																8,5
*Generalizability/translation*																		
Comment on whether, and how, the findings of this study are likely to translate to other species or systems, including any relevance to human biology	✓										✓							8.5
*Funding*																		
List all funding sources	✓	✓		✓	✓	✓	✓	✓	✓				✓	✓	✓	✓	✓	65.7
Results (%)	65.7	45.7	60.0	68.6	74.3	65.7	57.1	60.0	62.9	60.0	57.1	68.6	74.3	71.4	65.7	54.3	68.6	

## Supplementary Materials

Supplementary Materials Table S1: descriptors used in search platforms Pubmed and Scopus.
